# Does access to a portable ophthalmoscope improve skill acquisition in direct ophthalmoscopy? A method comparison study in undergraduate medical education

**DOI:** 10.1186/s12909-019-1644-5

**Published:** 2019-06-13

**Authors:** J. A. Gilmour-White, A. Picton, A. Blaikie, A. K. Denniston, R. Blanch, J. Coleman, P. I. Murray

**Affiliations:** 1 Institute of Clinical Sciences, University of Birmingham, Birmingham, UK; 2 Institute of Applied Health Research, University of Birmingham, Birmingham, UK; 3 Global Health Implementation Team, School of Medicine, University of St Andrews, Scotland, UK; 4 University Hospitals Birmingham NHSFT, Birmingham, UK; 5 Institute of Inflammation and Ageing, University of Birmingham, Birmingham, UK; 6 Academic Department of Military Surgery and Trauma, Royal Centre for Defence Medicine, Birmingham, UK

**Keywords:** Undergraduate medical education, Ophthalmology, Direct ophthalmoscopy

## Abstract

**Background:**

Direct ophthalmoscopy (DO) is an essential skill for medical graduates but there are multiple barriers to learning this. Medical students and junior doctors typically lack confidence in DO. Most students do not own an ophthalmoscope and learn via ward devices that vary in design and usability. The Arclight ophthalmoscope (AO) is an easy to use, low-cost and portable device that could help address device access. This study aimed to assess the impact of personal ownership of an AO on DO skill acquisition and competency amongst medical students in the clinical environment.

**Methods:**

Method comparison study with 42 medical students randomised to either traditional device ophthalmoscope (TDO) control or AO intervention group during an 18-week medical placement. Three objective assessments of DO competency were performed at the beginning and end of the placement: vertical cup to disc ratio (VCDR) measurement, fundus photo multiple-choice questions (F-MCQ) and model slide examination (MSE). DO examinations performed during the placement were recorded via an electronic logbook.

**Results:**

Students in both groups recorded a median number of six examinations each during an eighteen-week placement. There was no statistically significant difference between the groups in any of the objective assessment measures (VCDR *p* = 0.561, MCQ *p* = 0.872, Model *p* = 0.772). Both groups demonstrated a minor improvement in VCDR measurement but a negative performance change in F-MCQ and MSE assessments.

**Conclusions:**

Students do not practice ophthalmoscopy often, even with constant access to their own portable device. The lack of significant difference between the groups suggests that device access alone is not the major factor affecting frequency of DO performance and consequent skill acquisition. Improving student engagement with ophthalmoscopy will require a more wide-ranging approach.

## Background

Direct ophthalmoscopy is an essential skill for medical graduates as outlined by the General Medical Council (GMC) and supported by the Royal College of Ophthalmologists. [1, 2] Specific ophthalmic problems are estimated to make up approximately 1.46–6% of UK Emergency Department attendances and 1.5% of GP consultations. [3, 4] Timely and accurate DO can be life-saving in some patients, for example in recognising papilloedema. [5] DO is also required in the management of chronic multi-system diseases such as diabetes mellitus and hypertension.

Despite the importance of and frequent need to perform DO, there are multiple barriers to learning this skill at an undergraduate level. [6, 7] Ophthalmology is not a compulsory clinical attachment for all UK medical schools and consequently some students graduate without any ophthalmoscopy exposure. [8] Limited dedicated ophthalmic curricula time is a common finding globally affecting medical schools in both high and low resource countries. [9, 10] Perhaps unsurprisingly, cross-sectional studies highlight that medical students’ self-reported confidence in DO can be low. [10] These findings are continued after graduation, with UK studies of Foundation Year and ED doctors highlighting that the majority lack confidence using an ophthalmoscope correctly and in identifying pathology. [11, 12]

Another barrier to students to learning DO is limited assessments. Objective assessment of DO is difficult due to the inherent challenge that examiners cannot easily determine how well students can view a subject’s fundus. [13] Assessment drives learning behaviour and time-pressured medical students will inevitably prioritise knowledge and skills that they will be assessed on. A 2011 survey of UK medical schools highlighted that only 38% undertook formal assessment of students’ ophthalmoscopy skills. [8] Assessments and simulation models used may lack both objectivity and validity. [14]

Device access may be a major barrier to improving frequency of DO performance and associated skill acquisition. Most UK medical students do not own a direct ophthalmoscope or have easy access to a functioning device on hospital placements. Ownership of ophthalmoscopes amongst students fell dramatically following removal of equipment grants in 1986. [15] Subsequent students have therefore entered a learning environment where the norm is not to have their own device. The cost of a traditional direct ophthalmoscope (TDO) such as a Keeler standard model is around £220 and considered prohibitively expensive to most undergraduates. [16] Availability of ophthalmoscopes in hospital attachments is recognised to be limited. This is multi-factorial: NHS procurement can lack consistency in which models are purchased and ward staff may not provide ongoing maintenance leading to non-functioning devices due to burst bulbs or flat batteries. These issues present further challenges to skill mastery. [11]

**Fig. 1:**
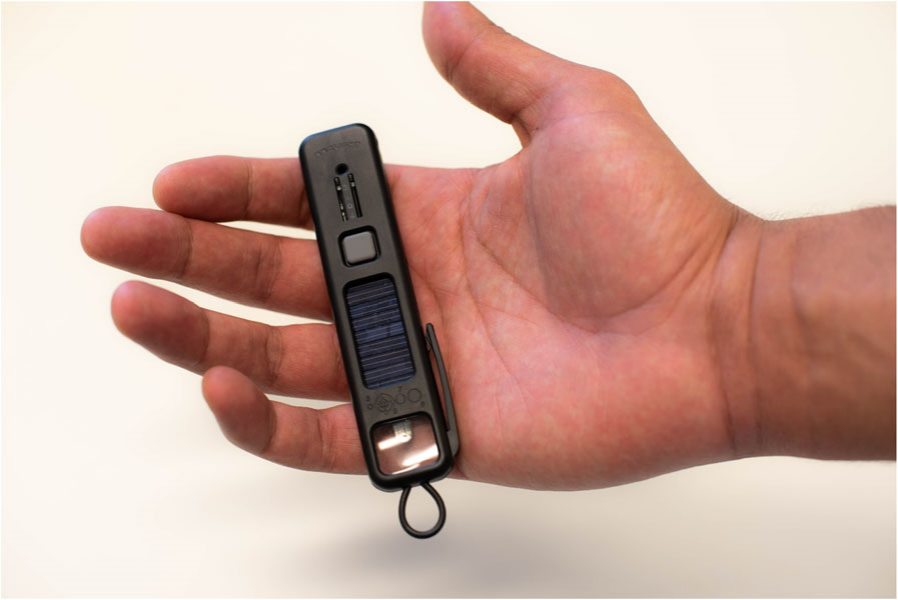
Arclight Ophthalmoscope

The Arclight (AO) is a device that offers promise in overcoming these barriers. It is a highly portable (11 cm long and weighing 18 g) solar powered, LED illuminated ophthalmoscope. In the UK it costs approximately £50, a significant reduction compared to TDOs. [17] (Fig. 1). Despite its low cost, previous studies have shown it to be as good as TDOs with the majority of users finding it easier to use. [17–19]

Consequently, the aim of this study is to assess the impact of personal ownership of a portable ophthalmoscope (AO) on DO skill acquisition and competency amongst medical students in the clinical environment compared to a control group with typical access to TDOs.

## Methods

### Design

We used a mixed methods design, primarily in the form of a method comparison study supported by a qualitative survey. Ethical approval was granted by the University of Birmingham ethics board in October 2016 (Refence: ERN_16–1021).

### Setting and participants

The study was performed amongst fourth year MBChB medical students at the University of Birmingham during the period November 2016 to April 2017. Participants were all undertaking their 18-week Specialty Medicine (SPM) hospital placement. SPM is a mandatory clinical attachment which involves rotation through different specialities including one to two weeks of ophthalmology. Students are randomly allocated between eight different hospitals across the West Midlands.

### Recruitment and randomisation

All 178 4th year medical students undertaking the SPM placement at the time of study recruitment were invited to participate via email. Students were offered a free AO for taking part in the study. The only additional eligibility criterion applied was that students were required to have a refractive error between -6D and + 4D to participate. This was to match the capacity of the AO to correct refractive error and is in keeping with previous studies. [17]

A total 42 students (24% response rate) were successfully recruited and individually randomised by the primary investigator (PI) using computerised random numbers to either the control or intervention arms. Three objective DO competency assessments were planned before the students started their 18-week SPM placement and were to be repeated at the end. The students in the intervention arm were given an AO to use throughout the study period and keep afterwards. Students in the control arm received their AO at the end of the study. All participants also then received individualised feedback in the form of their raw assessment scores. These were not graded or linked with any assessments within the MBChB programme.

### Control

Students randomised to the control group used TDOs during both the pre and post clinical attachment assessments. During their clinical attachment they only used the TDOs typically available in the hospitals of their SPM placements.

### Intervention

Students randomised to the intervention group all used their own personal AO during both assessments and their SPM placements. Students could replace lost or broken AOs by contacting the PI.

### Assessments

Three primary assessments of DO competency were performed on all participants at the study beginning and end: judgement of vertical cup disc ratio (VCDR), fundus multiple choice questions (F-MCQs) and model slide regional examination (MSE). VCDR, F-MCQ and EOU all necessitated performing ophthalmoscopy on other study participants, while MSE consisted of examining pre-generated fundal images on 35 mm slides in eye models. Students were all emailed information about the DO devices they would be using and the different assessments 2 weeks before the baseline assessment. No information was given on how to perform DO and no teaching was delivered on the day of assessments. Students were given ten minutes to familiarise themselves with their allocated device prior to the baseline assessments.

Students also self-assessed their examination competence for each ophthalmoscopy examination carried out on another study participant. This was via an ‘Ease of Use’ (EOU) scale used in a previous study, which ranged from 1 (‘Couldn’t use this ophthalmoscope’) to 8 (‘Determined a cup: disc ratio with a low level of difficulty). [20] This scale is included in Appendix 1.

### Model slide regional examination (MSE)

This assessment used fundus photo slides annotated with letters of various font sizes printed in different positions on the retina and placed within a mannequin (Eye Retinopathy Trainer®, Adam, Rouilly Co., Sittingbourne, UK). Each participant examined six model eyes each with six letters in the same pre-defined retinal locations but with reducing font sizes. Scores were calculated as a percentage total of the correct answers.

### Fundus photography

After recruitment, all participating students had fundus photographs taken of both their eyes by the PI using a Topcon® retinal fundus camera. These photographs were cropped to illustrate the optic nerve in the centre of an image with a one disc diameter surrounding area of retina and used to generate the F-MCQs.

### Fundus multiple choice questions (F-MCQs)

**Fig. 2:**
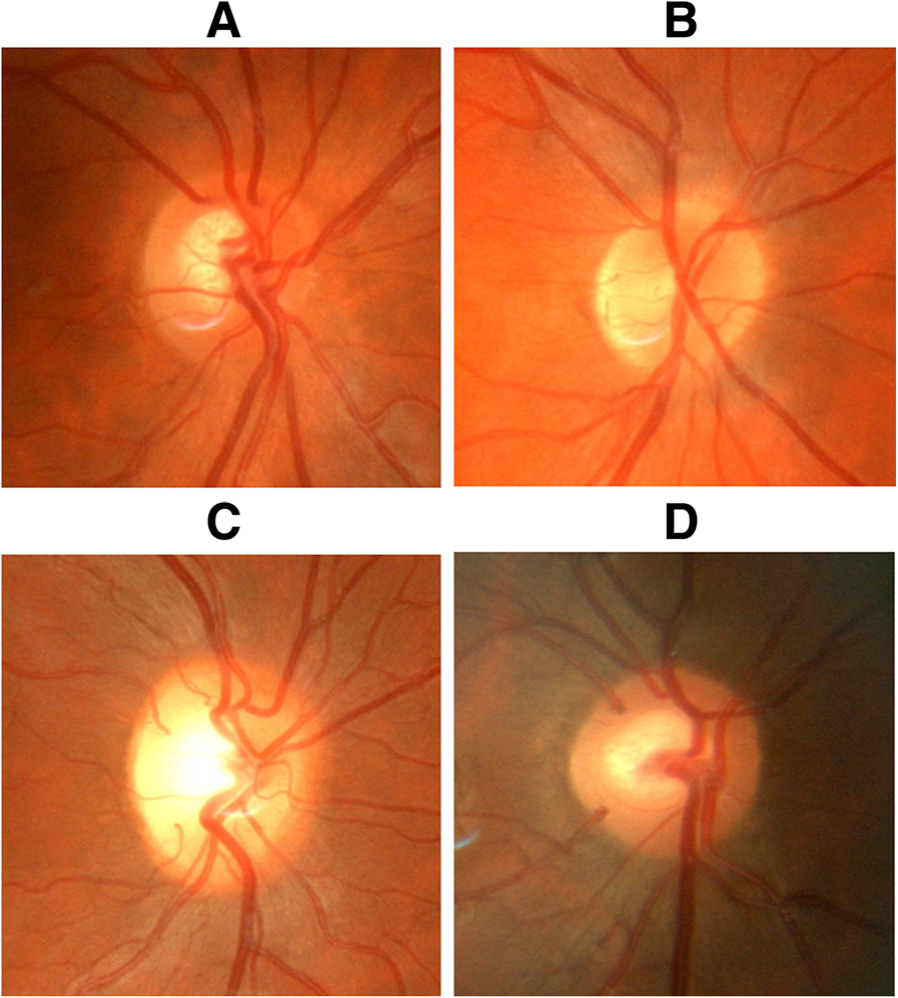
F-MCQ example

F-MCQ assessment sessions required every student to perform ophthalmoscopy on every other student. The examining student was required to identify the optic nerve of the student being examined. Specifically, each student had two F-MCQs (one for each eye) each with four images: their previously acquired optic nerve head image and three non-matching distractors from other participating students. See Fig. 2 for an example. One mark was awarded for a correct match and zero for an incorrect match.

### Vertical cup to disc ratio (VCDR)

Participants were requested to assess and record the VCDR of each eye examined. Three ophthalmic specialists (AB, RB and PIM) provided VCDR assessments for all optic nerve head images from the participants. The mean of these assessments was used to form the ‘gold standard’ from which participant results were compared. Students scored a mean magnitude error based on the comparison of each of their assessments to the gold standard.

### Electronic logbook

Students kept an electronic logbook (e-logbook) of all DO examinations they performed during their 18-week placement including EOU scores. Students coded this data during placement using a simple online application accessible via smart phones. Participants were contacted by email at six points during the study period and reminded to code examinations.

### Statistical analysis

Quantitative data was analysed according to a per protocol principle using the software SPSS Statistics (Version 24, IBM®). Comparison of baseline characteristics, including gender, refractive error and hospital placement was undertaken using Chi-squared test and Fisher’s exact test. Median/mean differences in DO competency were compared using the Wilcoxon Signed Rank Test for non-parametric and the Paired Samples t-test for parametric data. Intra class coefficients (ICCs) were used to measure the agreement of assessments in performance ranking participants. Correlations between performance and other independent factors were analysed using Spearman’s Rank Test.

## Results

**Fig. 3:**
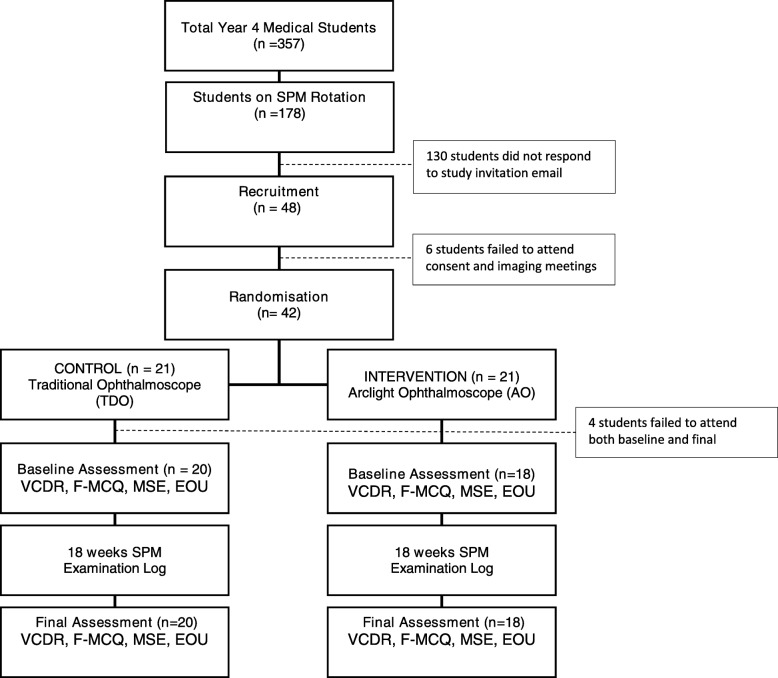
Study Flow Diagram

A total of 38 students (21% of cohort) completed the study (Fig. 3). Comparison of baseline characteristics including gender, refractive error and hospital placement demonstrated no statistically significant difference between the groups (Appendix 2).

The e-logbook demonstrated no difference in the median number of examinations performed by the AO group compared to control (6.0 vs 6.0) (Table 1). The greater mean number of examinations performed by the AO group vs control (9.6 vs 7.0, *p* = 0.41) was due to a small minority (*n* = 3) of students in the AO performing large numbers of examinations.

**Table 1:** E-logbook number of examinations

Group	Mean	Median	Minimum	Maximum
Control	7.0	6.0	1	28
Intervention	9.6	6.0	0	45
All	8.2	6.0	0	45

There was a minor reduction in the magnitude of VCDR judgement error in both groups; intervention − 0.12 (CI − 0.18 to − 0.05) vs control − 0.08 (CI − 0.15 to − 0.02) (Table 2). Both groups performed worse in the end assessments compared to their baseline assessments in F-MCQ and MSE assessments; intervention − 16.7 (IQR − 18.7 to 10.4, *p* < 0.01) vs control − 7.1 (IQR − 21.4 to − 1.8, *p* < 0.01) and intervention − 12.5 (IQR − 25 to 0, p < 0.01) vs control − 12.5 (IQR − 25 to − 12.5, p < 0.01) respectively. There was no statistically significant difference between these assessed competency changes (VCDR *p* = 0.561, MCQ *p* = 0.872, Model *p* = 0.772). The AO group demonstrated statistically significant increased EOU scores of 0.24 (CI 0.08 to 0.39) vs control 0.04 (− 0.14 to 0.24). Notably the AO also performed better at the F-MCQ assessments at baseline 58.3% vs control 42.9% (*p* = 0.013) and at final 45.8% vs control 35.7% (*p* = 0.043). There was no difference in scores between groups across the other assessment modalities. ICCs demonstrated no significant performance rank correlation between the assessments (VCDR/MSE 0.124, VCDR/F-MCQ -0.111, MSE/F-MCQ 0.096).

**Table 2:** Comparison of baseline and final outcome assessments

Assessment	Baseline (1) Mean (CI 95%) OR *Median (Q_1_-Q_3_)	Final (2) Mean (CI 95%) OR *Median (Q_1_-Q_3_)	Change (2–1) Mean (CI 95%) OR *Median (Q_1_-Q_3_)	*P* value
ALL VCDR (error)	0.55 (0.51 to 0.59)	0.45 (0.42 to 0.49)	-0.1 (−0.14 to − 0.05)	< 0.01
Control	0.56 (0.51 to 0.61)	0.48 (0.43 to 0.53)	−0.08 (− 0.15 to − 0.02)	< 0.01
Intervention	0.54 (0.48 to 0.56)	0.42 (0.37 to 0.48)	−0.12 (− 0.18 to − 0.05)	< 0.01
ALL F-MCQ (%)	*50 (35.7 to 66.7)	*41.2 (33.3 to 50.0)	*-8.3 (−21.4 to 0)	< 0.01
Control	*42.9 (37.2 to 62.8)	*35.7 (28.6 to 42.9)	*-7.1 (−21.4 to −1.8)	< 0.01
Intervention	*58.3 (33.3 to 77.1)	*45.8 (39.6 to 58.3)	*-16.7 (−18.7 to − 10.4)	< 0.01
ALL MSE (%)	*87.5 (75 to 100)	*75.0 (62.5 to 75)	*-12.5 (−25 to 0)	< 0.01
Control	*87.5 (75 to 100)	*75.0 (50 to 75)	*-12.5 (−25 to 0)	< 0.01
Intervention	*87.5 (75 to 100)	*75.0 (62.5 to 75)	*-12.5 (−25 to −12.5)	< 0.01
ALL EOU (score)	5.14 (5.03–5.24)	5.27 (5.16–5.38)	0.14 (0.01–0.26)	0.035
Control	5.15 (5.00–5.29)	5.19 (5.03–5.36)	0.04 (−0.14 to 0.24)	0.624
Intervention	5.12 (4.98–5.26)	5.36 (5.22–5.55)	0.24 (0.08 to 0.39)	0.03

## Discussion

The key finding from our study was the low numbers of DO examinations performed by both groups; median of six during the 18-week clinical attachment which included 1 to 2 weeks of ophthalmology. The low number of examinations is particularly striking given that participants self-selected for study involvement, knew they were being observed and the intervention group were given free portable ophthalmoscopes. Students may have simply failed to record examinations, although this seems unlikely given the potentially positive effect of observer bias and easy access to the smartphone-based e-logbook.

A limitation of studies in this field of research is a lack of a validated objective measure of ophthalmoscopy skills at an undergraduate level. We chose a range of assessments to provide an overview of student performance in an attempt to overcome this. VCDR and EOU scoring [17, 20], F-MCQ [13, 21] and MSE [22, 23] have all been used in similar studies before but not directly compared or formally validated for assessing competence.

Similar competency results were observed between intervention and control groups across all three assessments. Both groups demonstrated a minor improvement in VCDR judgement but a reduction in F-MCQ and MSE performance. Students generally found VCDR assessment challenging, which is not surprising given the significant assessment variation even amongst ophthalmic specialists. [24] Given the lack of correlation with number of examinations, the minor improvement seen in VCDR judgement was likely due to general ophthalmology placement experiences or personal study rather than DO practice. (Appendix 3) Our results suggest F-MCQs may show promise going forwards as they were the only assessment modality to positively correlate with the number of examinations performed. (Appendix 3).

Anecdotally, students reported finding the second set of MSE slides harder to visualise. This was confirmed by the PI and was likely due to variation in the print quality or letter type. The reduction in performance scores in the final MSE assessments may have been in part due to this. This was not the case for F-MCQs as the same questions were used at both baseline and final assessment. MSE has inherent limited construct validity and in our study appeared to be affected by variances in difficulty. There was also a significant correlation with refractive error i.e. students with greater refractive error performed worse in MSE assessments than their peers, which suggests this is a source of performance bias for MSE.

VCDR, F-MCQ and MSE appeared to be testing different aspects of DO competency. This is supported by a lack of significant intra class coefficient (ICC) between any of the assessments. Further research is required to develop a fit for purpose objective measure for DO competency at the undergraduate level.

The AO may provide some performance advantage over traditional models. Despite the lack of impact of the AO on number of examinations and DO skill acquisition, our study confirmed non-inferior performance of the AO versus TDO in 2 of the 3 objectively assessed modalities and higher F-MCQs scores at both the baseline (58.3% vs 42.9%) and final assessments (45.8% vs 35.7%). Furthermore, there was a statistically significant increase in self-assessed EOU score for students using the AO.

Further research should aim to explore students’ attitudes towards and experience of practising ophthalmoscopy to help identify what barriers to DO skill acquisition are present at an undergraduate level and how to address these. One factor not addressed by this study is clinical supervision and availability of experienced supervisors. Junior doctors often provide frontline clinical teaching but if they lack confidence in their own ophthalmoscopy skills this may lead to a reluctance to support and guide students. [11]

### Strengths and limitations

The strengths of this study were randomising students into a control group and intervention group, use of novel technology and collection of longitudinal data on clinical attachment combined with assessment data. We acknowledge the following limitations:

- This research was carried out at one institution only so will reflect the curriculum and clinical experience available.
- The study may be underpowered due to a relatively small analysed sample size (*n* = 38). Without any similar previous or pilot studies it was not possible to perform a reliable power calculation.
- Due to the nature of the intervention, it was not possible to mask either the educators or students to which device was being used by each group.
- Students’ ophthalmology week took place during any one of the 18 weeks of SPM attachment and we did not record when this took place for individual students. To what degree the timing of this week affected results is unknown. For example, students who had their ophthalmology week first may have been more confident performing ophthalmoscopy in the rest of the block and vice versa.
- The assessment measures lacked validation, particularly the F-MCQs. For each F-MCQ distractor images were picked to provide contrast for example different vasculature or VCDR but this limited standardisation and questions may have varied in difficulty.
- E-logbook data was self-reported. Students may have under-reported or entered false examinations.

## Conclusions

In our study, personal ownership of a portable ophthalmoscope offered limited advantage over traditional models. Students did not practice DO frequently, even with access to their own portable device. This was reflected in a lack of any meaningful improvement in DO skill over the study period. The AO represents a suitable alternative to more expensive traditional devices, but our results suggest changing student engagement with ophthalmoscopy will require a more wide-ranging approach than improving device access alone.

## Data Availability

The datasets used and/or analysed during the current study are available from the corresponding author on reasonable request.

## Appendix 1

**Table 3:** EOU Score

Ease Of Use (EOU) Score	
[1] Could not use at all	
[2] Could not see the red reflex to even begin with	
[3] Could identify red reflex	
[4] Could see vessels but not disc	
[5] Could identify disc but not vertical CD-ratio (VCDR)	
[6] Could determine VCDR with a high level of difficulty	
[7] Could determine VCDR with a medium level of difficulty	
[8] Could determine VCDR with a low level of difficulty	

## Appendix 2

**Table 4:** Baseline Characteristics

	Control (*N* = 21) % or Median (Q_1–3_)	Intervention (*N* = 21) % or Median (Q_1–3_)	p
Percent female	67	76	0.495
Percent emmetropic	57	38	0.217
Refractive error	0 (−2 to 0)	0.5 (−1.8 to 0)	0.658
Percent placement			
Hospital 1	38	43	0.753
Hospital 2	9.5	19	0.378
Hospital 3	5	14	0.293
Hospital 4	19	14	0.679
Hospital 5	9.5	5	0.549
Hospital 6	5	0	0.311
Hospital 7	14	0	0.072
Hospital 8	0	5	0.311

## Appendix 3

**Table 5:** Independent Factor Correlations

		Intervention	Gender	Examinations	Refraction	Hospital
VCDR 2–1	Correlation Coefficient	−.042	.063	.166	.073	−.073
	Sig. (2-tailed)	.361	.176	.000	.114	.114
	N	466	466	466	466	466
F-MCQ 2–1	Correlation Coefficient	.007	.045	.094	−.001	−.010
	Sig. (2-tailed)	.872	.330	.043	.975	.836
	N	466	466	466	466	466
MSE 2–1	Correlation Coefficient	−0.19	.018	.089	−.146	.114
	Sig. (2-tailed)	.772	.782	.182	.027	.087
	N	228	228	228	228	228
EOU 2–1	Correlation Coefficient	.108	.057	−.090	−.065	.034
	Sig. (2-tailed)	.020	.222	.051	.164	.468

*VCDR* – Vertical Cup Disc Ratio

*F-MCQ* – Fundus Multiple Choice Question

*MSE* – Model Slide Examination

*EOU* – Ease of Use Score

## Abbreviations

AO: Arclight ophthalmoscope
DO: Direct ophthalmoscopy
EOU: Ease of use score
F-MCQ: Fundus multiple choice question
ICC: Intra class coefficient
MSE: Model slide examination
SPM: Speciality medicine
TDO: Traditional direct ophthalmoscope
VCDR: Vertical cup disc ratio
